# A Cellular Automata Model of Oncolytic Virotherapy in Pancreatic Cancer

**DOI:** 10.1007/s11538-020-00780-5

**Published:** 2020-07-31

**Authors:** J. Chen, D. Weihs, F. J. Vermolen

**Affiliations:** 1 Department of Biomedical Engineering and Physics, Amsterdam UMC, Amsterdam, The Netherlands; 2 Delft Institute of Applied Mathematics, Delft University of Technology, Delft, The Netherlands; 3 Faculty of Biomedical Engineering, Technion-Israel Institute of Technology, Haifa, Israel; 4 Division of Mathematics and Statistics, Faculty of Sciences, Hasselt University, Diepenbeek, Belgium

**Keywords:** Cellular automata, Computational modeling, Cancer treatment, Virotherapy, Monte Carlo simulations

## Abstract

Oncolytic virotherapy is known as a new treatment to employ less virulent viruses to specifically target and damage cancer cells. This work presents a cellular automata model of oncolytic virotherapy with an application to pancreatic cancer. The fundamental biomedical processes (like cell proliferation, mutation, apoptosis) are modeled by the use of probabilistic principles. The migration of injected viruses (as therapy) is modeled by diffusion through the tissue. The resulting diffusion–reaction equation with smoothed point viral sources is discretized by the finite difference method and integrated by the IMEX approach. Furthermore, Monte Carlo simulations are done to quantitatively evaluate the correlations between various input parameters and numerical results. As we expected, our model is able to simulate the pancreatic cancer growth at early stages, which is calibrated with experimental results. In addition, the model can be used to predict and evaluate the therapeutic effect of oncolytic virotherapy.

## Introduction

**Fig. 1:**
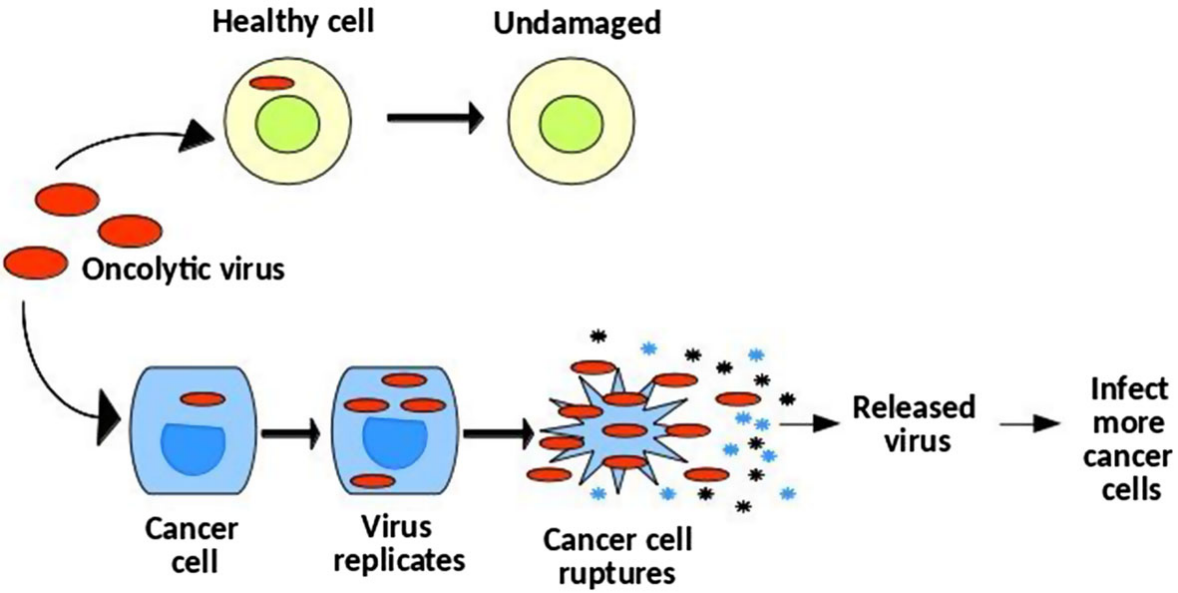
A schematical figure of oncolytic virotherapy. The viruses can specifically infect cancer cells and then replicate themselves until cancer cells rupture. Subsequently, the newborn viruses are released to infect more cancer cells

**Fig. 2:**
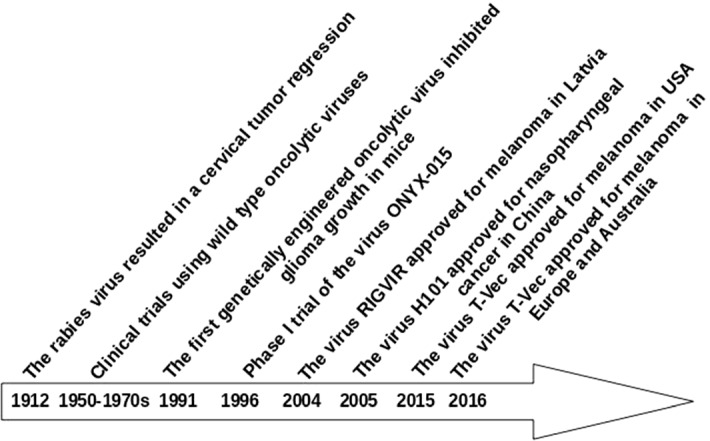
Historical milestones in the development of oncolytic virotherapy

Oncolytic virotherapy is a novel cancer treatment where natural or genetically modified viruses infect cancer cells and then self-replicate until the host cancer cells lysis (see Fig. 1). Ruptured cancer cells release chemicals like tumor antigens, which make cancer cells easily recognizable by the immune system. Moreover, the released viruses can infect more cancer cells to trigger a chain reaction and effectively act as a follow-up treatment. As early as in 1912, De Pace (1912) observed a tumor regression after inoculation of an attenuated rabies vaccine in a patient with uterine cervical carcinoma. Later on, an animal-based test (Levaditi and Nicolau 1922) and a human trial (Pack 1950) were conducted in 1920 and 1940, respectively, where both experiments yielded an obvious partial tumor regression (Kasuya et al. 2005). In the subsequent decades, more works (Kirn 2001; Gil et al. 2013, 2014) demonstrated that oncolytic virotherapy lead to tumor attenuation. Some milestones in the development of oncolytic virotherapy are shown in Fig. 2 (De Pace 1912; Martuza et al. 1991; Xia et al. 2004; Fukuhara et al. 2016).

Pancreatic ductal adenocarcinoma (PDA), recognized as the most common pancreatic cancer, is a lethal disease due to late detection, a low resectability rate, medication resistance and poor prognosis (Korc 2007; Gore and Korc 2014). Currently, pancreatic cancer is the seventh leading cause of cancer death worldwide and its 5-year survival rate is less than 5% (Feig et al. 2012; Bray et al. 2018). Compared with other types of cancer, PDA has more cancer-associated fibroblasts (CAFs) resulting in abundantly desmoplasitic stroma that constitutes up to 90% of a solid tumor volume (Moir et al. 2015; Öhlund et al. 2017). The profuse desmoplasia in stroma produced by CAFs acts as a physical barrier for drug delivery and leads to medication resistance (Provenzano et al. 2012; Jacobetz et al. 2013). However, CAFs make cancer cells more susceptible to be infected by oncolytic viruses. Ilkow et al. (2015) experimentally demonstrated that the cross-talk between cancer cells and CAFs facilitates the oncolytic virus-based therapies. Therefore, oncolytic virotherapy offers an avenue for the treatment of pancreatic cancer.

The ideal oncolytic virus should be able to selectively replicate itself in cancer cells without damaging normal somatic cells (see Fig. 1). A couple of studies (Sunamura et al. 2004; Kasuya et al. 2005; Wennier et al. 2011) summarized advantages and disadvantages of various replication-competent oncolytic viruses proposed for pancreatic cancer therapies, e.g., adenoviruses, herpesviruses, poxviruses, parvoviruses, reoviruses and paramyxoviruses. A few types of viruses have been tested in animal-based xenograft models; however, only a few kinds of viruses have reached clinical trials. In particular for pancreatic cancer, relevant studies are rare, among which Fu et al. (2006) observed that a type of oncolytic virus produced antitumor effects in human pancreatic cancer xenografts. In addition, Sunamura et al. (2004) carried out adenovirus therapy in immunodeficient mice with human pancreatic cancer xenografts that resulted in a remarkable inhibition of tumor growth under consecutive injections of the virus. Typically, if animal testing is successful, the new drug will reach clinical trials that are classified into four phases: (1) phase I is a safety test for healthy volunteers; (2) phase II demonstrates whether a drug can have any efficacy against the disease; (3) phase III checks in a randomized multicenter tests if a drug has the right therapeutic effect; and (4) phase IV post-marketing surveillances its efficacy and side effects after extensive use. Regarding the clinical applicability, Kasuya et al. (2005) stated that a clinical trial of viruses adenovirus ONYX-015 (phase I and II) has been conducted in pancreatic cancer patients, where half of the patients (phase II) exhibited either tumor reduction or stabilization. In contrast, a phase I trial of the efficacy of several oncolytic herpes viruses (such as G207, 1716 and OncoVEX GM-CSF) have tested against various tumors and the herpesvirus exhibited a good tolerance at all dosages. Although oncolytic virotherapy has been proposed for decades, a thorough understanding of the interactions of virus, tumor and microenvironment in vivo is still needed to be further researched, like a proper viral dose for a specific virus, how to control of virulence and etc. Therefore we develop a three-dimensional (3D) spatial Markov Chain cellular automata model to mimic pancreatic tumor (pancreatic cancer at early stages) progression and subsequent regression under the interference of oncolytic virotherapy. In addition, the model can be used to quantify the impact of virotherapy with different viral doses, viral infectivity and levels of immunity in patients with pancreatic cancer.

Cellular automata models are lattice-based models that facilitate analysis of the spatiotemporal dynamics based on the interplay between cells and their microenvironment. The cellular automata model has been introduced as a computer model of self-reproduction by John von Neumann and Stanislaw Ulam (Langton 1984). In the past decades, cellular automata models, in addition to self-reproduction, have been extended to other model applications successfully within a wide range of spectrum from biology, physics, chemistry to other sciences (Deutsch et al. 2005). Regarding the cancer modeling in cellular automata, Reis et al. (2009) proposed a model that could capture the Gompertzian behavior of tumor growth. Hatzikirou et al. (2008) developed a model of tumor invasion dynamics. In addition, a couple of studies demonstrated applications of cellular automata in cancer therapy, e.g., radiotherapy (Enderling et al. 2010), and chemotherapy (Pourhasanzade and Sabzpoushan 2019). In terms of computational models on oncolytic virotherapy, Wodarz et al. (2012) studied the distinct patterns of oncolytic viral spreading through tumor cell population both experimentally and computationally. Furthermore, Paiva et al. (2009) developed a multiscale cellular automata model for oncolytic virotherapy, where authors found that a solid tumor can be either eradicated completely or keeps on growing. The resulting behavior depends on the input parameters, which represent the biological circumstances around the tumor. Malinzi et al. (2017) developed a partial differential equation-based model for oncolytic virotherapy. For the one-dimensional case, they assessed the stability of traveling wave solutions and the rate of tumor progression by means of the minimal traveling wave speed. One of their most important conclusions is that the combination of chemotherapy and virotherapy most successfully removes tumors.

The 2D model in Paiva et al. (2009) revealed an oscillatory behavior of cancer cells and virus population, which hints a strong host immune response is necessary. As an extension of this model, we develop a 3D model to phenomenologically show pancreatic cancer initiation at early stages and its subsequent regression under oncolytic virotherapy, where the various levels of host immune responses and toxicities of residual viruses are taken into account. To the best of our knowledge, it is the first description of a hybrid cellular automata model with an application to pancreatic cancer. In addition, this model also shows the pattern of virus spreading by using partial differential equations in a spatial domain. One of the advantages is its efficacy in evaluation of therapeutic outcomes based on injected dose of viruses, viral infection potent and personal immune strength. Next to the development of the model, we calibrate the model to experimental outcomes and we carry out an uncertainty quantification by using Monte Carlo simulations in terms of assessment of the likelihood of success of the treatments. Therefore, therapeutic effects as well as toxicities of residual viruses are predicted, which is expected to be helpful for viral administration and the patient-specific treatment.

## Mathematical Formalism

Cellular automata models consist of a class of lattice-based models, where lattice approaches are classified as: (1) a single lattice site is occupied by one cell only; (2) a single lattice site is occupied by a cluster of cells; (3) one cell takes many lattice sites. They are all capable of investigating biological processes with single cell or multiple cells, where division, death or other biomedical phenomena are modeled by stochastic processes (Van Liedekerke et al. 2015). In contrast, the first two categories are typically used to describe volume effects, whereas the last category is able to capture the morphological evolution of cells.

In our simulations, each lattice represents a volume element filled with multiple cells in a bounded 3D computational domain ${\Omega } \subset \mathbb {R}^3$, which is divided into a set of lattice points $N = \{1,\ldots , n\}$. The boundary of ${\Omega }$ is denoted by ${\Gamma }$. The lattice point *i* has a finite number of discrete states $S_i$ that indicates the state of cells in the corresponding volume, which reads as

1 $$\begin{aligned} S_i = {\left\{ \begin{array}{ll} 0, \quad \text {lattice point }i\text { is in unoccupied state/necrotic cell state}\\ 1, \quad \text {lattice point }i\text { is in epithelial cell state}\\ 2, \quad \text {lattice point }i\text { is in uninfected cancer cell state}\\ 3, \quad \text {lattice point }i\text { is in infected cancer state}\\ \end{array}\right. }. \end{aligned}$$

**Fig. 3:**
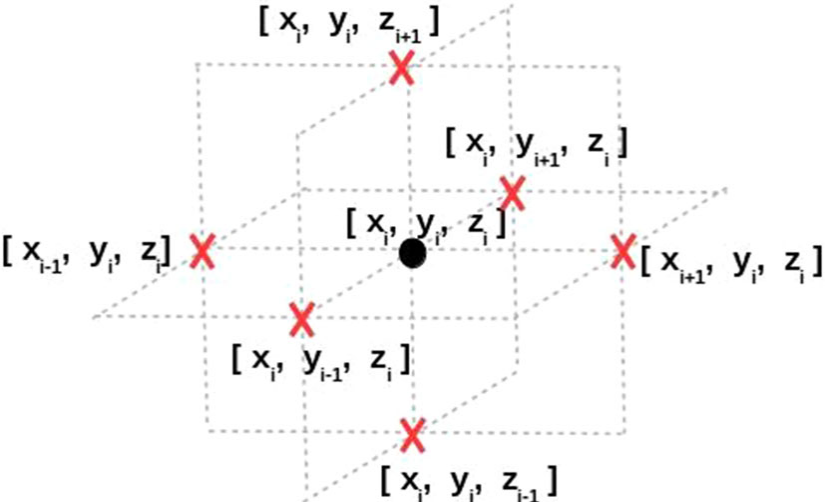
A specified lattice *i* at position $\mathbf{x}_i= [x_i, y_i, z_i]$ with its neighborhood in the 3D simulations

Assigning an initial state for each lattice point, and subsequently adjusting the state of the specified lattice at position $\mathbf{x}_i= [x_i, y_i, z_i]$ at subsequent times is correlated with the states of its neighborhood marked in Fig. 3.

Subsequently, we consider fundamental biological processes like cell division, mutation, infection and death modeled as stochastic processes. The likelihood of changing a state of a lattice *i* to another state satisfies a memoryless exponential distribution which is given by $f(\lambda _i, t){\Delta } t$ during a time period $(t_0, t_0+{\Delta } t)$. Here $\lambda _i$ is the probability rate per unit of time, which is determined by biological mechanisms like cell division, mutation, infection and death. The $f(\lambda _i, t)$ reads as,

2 $$\begin{aligned} f(\lambda _i, t) = \lambda _i \mathrm {exp}(-\lambda _i(t-t_0)), \end{aligned}$$

and hence the transition probability *P* within a time interval of length ${\Delta } t$ is given by

3 $$\begin{aligned} P = \int _{t_0}^{t_0 + {\Delta } t} f(\lambda _i, t)\mathrm {d}t \simeq 1-\mathrm {exp}(-\lambda _i {\Delta } t). \end{aligned}$$

In the work by Vermolen and Pölönen (2020), it is proved that the likelihood of changing state depends on a simple binary states of the neighbors, which has been applied to modeling the progression of skin cancer. All lattice points in the domain are initialized to the epithelial cell state $\{S_i = 1\}$ or unoccupied state $\{S_i = 0\}$. During the growth of epithelial cells, cancer mutation happens as a result of exposure to carcinogenic factors like genetic inheritance, chemical carcinogens, electromagnetic radiation, or viral infection. The very complicated biological process is simplified by the application of a transition probability from epithelial cells to uninfected cancer cells, that is from $\{S_i = 1\}$ to $\{S_i = 2\}$ over a time interval ${\Delta } t$ with a likelihood following

4 $$\begin{aligned} {\left\{ \begin{array}{ll} P(S_i(t_0 + {\Delta } t) = 2 \mid S_i(t_0) = 1) \simeq 1-\mathrm {exp}(-\lambda _{\mathrm {mu}} {\Delta } t)\\ P(S_i(t_0 +{\Delta } t) = 1 \mid S_i(t_0) = 2) = 0 \end{array}\right. }. \end{aligned}$$

Here $\lambda _{\mathrm {mu}}$ represents the mutation probability rate per unit of time and the second part of Eq. (4) reflects that this transition is irreversible. Since both epithelial cells and cancer cells are able to proliferate and to migrate, lattice points are allowed to change their states from $\{S_i = 0\}$ to $\{S_i = 1,2\}$. The likelihood for these transitions over a time interval ${\Delta } t$ are given by

5 $$\begin{aligned} P(S_i(t_0 + {\Delta } t) = \{1, 2\} \mid S_i(t_0) = 0) \simeq 1-\mathrm {exp}(-\lambda _{\mathrm {pro}} {\Delta } t), \end{aligned}$$

where $\lambda _{\mathrm {pro}}$ denotes the probability rate of transition from ‘not occupied by any cells’ to being ‘occupied by either epithelial cells or uninfected cancer cells’. Note that the probability rate $\lambda _{\mathrm {pro}}$ is determined by the number of neighbors that are in cancer/ epithelial cell state. That is $\lambda _{\mathrm {pro}} = \lambda _{\mathrm {max}}\frac{n_{12}}{h}$, where $n_{12}$ denotes the number of lattice points that are either in state 1 or in state 2. The distance between two lattice points is represented by *h* and $\lambda _{\mathrm {max}}$ is a constant to regulate the overall growth rate of cells (including epithelial and uninfected cancer cells). Whether a free lattice point will be occupied by multiple epithelial cells or uninfected cancer cells depends on the states of the surrounding lattice points. Consider an unoccupied node *i* at time *t*, that is $S_i(t) = 0$. We denote the number of neighboring lattice points that are in state 1 by $n_1$. We further denote the likelihood that the lattice point *i*, given that it changes state, by $\alpha = \alpha _0 \cdot \frac{n_1}{n_{12}}$, where $\alpha _0 \in [0, 1]$ is a constant. This fraction $\alpha$ is used to determine the transition probability of node *i*, which is given by

6 $$\begin{aligned} {\left\{ \begin{array}{ll} P(S_i(t_0 + {\Delta } t) = 1 \mid S_i(t_0) = 0) = \alpha P(S_i(t_0 + {\Delta } t) = \{1, 2\} \mid S_i(t_0) = 0)\\ P(S_i(t_0 + {\Delta } t) = 2 \mid S_i(t_0) = 0) = (1-\alpha ) P(S_i(t_0 + {\Delta } t) = \{1, 2\} \mid S_i(t_0) = 0) \end{array}\right. }. \nonumber \\ \end{aligned}$$

Apoptosis is programmed death of cells, however, uninfected cancer cells are able to proliferate uncontrollably and to resist cell apoptosis. In the current model, oncolytic viruses are incorporated to infect and damage cancer cells. We let cancer cells jump from state $\{S_i = 2\}$ (uninfected cancer state) to state $\{S_i = 3\}$ (infected state) as soon as the virus concentration exceeds $\hat{c}$. Hence, we have

7 $$\begin{aligned} P(S_i(t_0 + {\Delta } t) = 3 \mid S_i(t_0) = 2) = 1, \quad \mathrm {if} \ c_i \>\hat{c}, \end{aligned}$$

If the viral concentration does not exceed the threshold $\hat{c}$, then we disregard the release of viruses. Therefore, the likelihood of a lattice point *i* to be infected by viruses, which is the state transition from $\{S_i = 2\}$ to $\{S_i = 3\}$, depends on the released viruses from the neighborhood and the local concentration of viruses. Subsequently, infected cancer cells are, like epithelial cells, subject to possible cell death. Hence a node *i* is allowed to change from a cell state ${\{S_i = \{1, 3\}\}}$ to an unoccupied state $\{S_i = 0\}$, which is given by the following likelihood

8 $$\begin{aligned} P(S_i(t_0 + {\Delta } t) = 0 \mid S_i(t_0) = \{1,3\}) \simeq 1-\mathrm {exp}(-\lambda _{\mathrm {de}} {\Delta } t). \end{aligned}$$

Here $\lambda _{\mathrm {de}}$ denotes the probability rate that an infected cancer or an epithelial cell dies.

Oncolytic virotherapy is initiated when the fraction of tumor constitutes up to 100% of the computational domain (that is the tissue). In animal-based experiments, the viruses are given by injections (Sunamura et al. 2004). Therefore, we consider one or multiple injections, denoted by $\mathbb {V}(t)$, as source points at position $\mathbf{x}_p$ by using the Dirac delta function $\delta (\mathbf{x})$ at time *t*. After the injection, the delivery of viruses is simulated by the reaction-diffusion equation written as

9 $$\begin{aligned} {\left\{ \begin{array}{ll} \frac{\partial c}{\partial t}- D{\Delta } c =\displaystyle \sum _{p \in \mathbb {V}(t)}\gamma \delta (\mathbf{x}-\mathbf{x}_p) + u(\mathbf{x}), &{} { \ \text {for} \ \mathbf{x} \in \ {\Omega }}\\ D\frac{\partial c}{\partial n} + T c = 0, &{} \quad \mathrm {on} \ \partial {\Gamma } \end{array}\right. }, \end{aligned}$$

where *c*, *D* and $\gamma$ denote the concentration, diffusivity and injection rate of virus in this domain. Note that $u(\mathbf{x})$ is utilized to model an increase in production of viruses released by necrotic cancer cells. Moreover, *T* represents the mass transfer rate coefficient between the computational domain ${\Omega }$ and its environment. Since viruses infect cancer cells and copy themselves until host cells lysis, infected cancer cells act as sources where newborn viruses originate. We define $\Omega _{\mathrm {ic}}(t)$ to denote the portion of the computational domain that is occupied by virally infected cancer cells. The function $u(\mathbf{x})$ in Eq. (9) increases as

10 $$\begin{aligned} u(\mathbf{x}) ={\left\{ \begin{array}{ll} \beta c (1-\frac{c}{N_v}), &{}\quad \mathrm {if} \ \mathbf{r}\ \in \ \Omega _{ic}(t) \\ 0, &{} \quad \mathrm {else} \end{array}\right. }, \end{aligned}$$

where $\beta$ and $N_v$ denote the proliferation rate of virus and a burst size of viruses from a necrotic cancer cell. The function $u(\mathbf{x})$ is a hypothetic function that accounts for the regeneration of virus particles as long as the cancer cell has been infected. We assume that the carrying capacity of the viral particles is determined by the availability of limited amounts of resources in the cancer cell. This assumption translates into the above logistic differential equation. This function can be revised easily if experimental observations require this.

Since most clinical data are not available and some parameters have even never been measured, we estimate some of the input parameters, which are listed in Table 1.

**Table 1:** Input values

Parameters	Notation	Value and Units	Source
Virus diffusivity	*D*	0.01 $\mathrm {mm}^2/\mathrm {h}$	Bajaj et al. (2001)
Injection rate	$\gamma$	1E4 $\mathrm {pfu}/(\mathrm {mm}^3\cdot \mathrm {h})$	Aghi and Martuza (2005)
Probability rate of cell mutation	$\lambda _{\mathrm {mu}}$	5 $\mathrm {h}^{-1}$	Estimated
Probability rate of cell death	$\lambda _{\mathrm {de}}$	5 $\mathrm {h}^{-1}$	Estimated
New burst size of viruses	$N_v$	100	–
Viral infection threshold	$\hat{c}$	$10~\mathrm {pfu}/\mathrm {mm}^3$	Estimated

## Numerical Method

### Discretization

We first consider a 2D or 3D that is occupied by uninfected cancer cells, where a dose of oncolytic viruses is injected into the computational domain as part of the therapy. Subsequently, viruses diffuse and thereby spread throughout the domain. The change in concentration of viruses is modeled by the reaction diffusion equation

11 $$\begin{aligned} {\left\{ \begin{array}{ll} \frac{\partial c}{\partial t} = D {\Delta } c + \gamma \delta (\mathbf{x} - \mathbf{x}_p) + \beta c (1-\frac{c}{N_v}),&{}\quad { \ \text {for} \ \mathbf{x} \in \ {\Omega }}\\ D\frac{\partial c}{\partial n} + T c = 0, &{}\quad \mathrm {on} \ \partial {\Gamma } \end{array}\right. }. \end{aligned}$$

Note that the above equation is a combination of Eqs. (9) and (10), in which we only take one injection source into account. The numerical method is described for the case of only one injection source since our simulations are only carried out for one source only. We note that incorporating multiple injection sources would not change the numerical method conceptually.

To solve the problem in 2D, the Laplace operator is discretized by the finite difference method (FDM) as,

12 $$\begin{aligned}&{\Delta } c(x,y,t) \nonumber \\&\quad \simeq \frac{c(x+h, y,t) + c(x-h, y,t) + c(x, y+h,t) + c(x, y-h,t) - 4c(x,y,t)}{h^2}, \end{aligned}$$

where *h* is the distance between adjacent lattice points. If the computational domain is extended to 3D, the above equation needs to be revised to

13 $$\begin{aligned} \begin{aligned}&{\Delta } c(x,y,z,t) \\&\quad \simeq \frac{1}{h^2}(c(x+h, y, z,t) + c(x-h, y, z,t) + c(x, y+h, z,t) + c(x, y-h,z,t)\\&\quad \quad + c(x, y,z+h,t) +c(x, y,z-h,t) - 6c(x,y,z,t)). \end{aligned} \end{aligned}$$

If a lattice point on boundary ${\Gamma }_{x = 0}$, then the point $(-h,y,z)$ is assumed as a virtual point out of the computational domain. The Robin boundary condition in Eq. (11) is dealt with the FDM as,

14 $$\begin{aligned} \frac{c(-h, y, z, t) - c(h, y, z,t) }{2h} = -\frac{T}{D}c(0, y, z), \end{aligned}$$

and thereby the viral concentration at the virtual point is calculated by,

15 $$\begin{aligned} c(-h, y, z, t) = c(h, y, z, t)- c(0, y, z, t)\left( 1-\frac{2T}{D}h\right) . \end{aligned}$$

Analogously, the viral concentration on the other virtural points can be obtained by

16 $$\begin{aligned} {\left\{ \begin{array}{ll} &{}c(x, -h, z, t) = c(x, h, z)-c(x, 0, z, t)\left( 1-\frac{2T}{D}h\right) , \ \mathrm {on} \ {\Gamma }_{y = 0}\\ &{}c(x, y, -h, t) = c(x, y, h)-c(x, y, 0, t)\left( 1-\frac{2T}{D}h\right) , \ \mathrm {on} \ {\Gamma }_{z = 0} \end{array}\right. }. \end{aligned}$$

Similarly, if a lattice point is located on boundary ${\Gamma }_{x= x_n}$, ${\Gamma }_{y= y_n}$ or ${\Gamma }_{z= z_n}$, the viral concentration at the corresponding virtual point is estimated by

17 $$\begin{aligned} {\left\{ \begin{array}{ll} c(x_n+h, y, z, t) = c(x_n-h, y, z, t)-c(x_n, y, z, t)\left( 1-\frac{2T}{D}h\right) , &{}\quad \mathrm {on} \ {\Gamma }_{x = x_n}\\ c(x, y_n+h, z, t) = c(x, y_n-h, z, t)-c(x, y_n, z, t)\left( 1-\frac{2T}{D}h\right) , &{} \quad \mathrm {on} \ {\Gamma }_{y = y_n}\\ c(x, y, z_n+h, t) = c(x, y, z_n-h, t)-c(x, y, z_n, t)\left( 1-\frac{2T}{D}h\right) , &{}\quad \mathrm {on} \ {\Gamma }_{z = z_n} \end{array}\right. }. \end{aligned}$$

Furthermore, the injection of viruses is simulated by a point source that is mathematically inspired by the Dirac Delta function $\delta (\mathbf{x})$, which is mollified by using the normal distribution,

18 $$\begin{aligned} \delta \left( \mathbf{x}\mid \mathbf{x}_p,\varepsilon ^2 \right) = \left( \frac{1}{2\pi \varepsilon ^2}\right) ^{d/2}\mathrm {exp}\left( -\frac{\parallel \mathbf{x}-\mathbf{x}_p\parallel ^2}{2\varepsilon ^2}\right) , \end{aligned}$$

where $\varepsilon$ and *d*, respectively, denote the source width and the dimensionality.

### Time Integration

To update the concentration of virus on each lattice at the next time step, an IMplicit-EXplicit(IMEX) time integration is utilized, where the linear parts and nonlinear parts are treated by a Euler backward method and a Euler forward method, respectively. Thereby the concentration of virus *c* is updated by

19 $$\begin{aligned} c^{n+1} = c^n + {\Delta } t\left( D {\Delta } c^{n+1} +{\gamma }\delta \left( \mathbf{x} - \mathbf{x}_p\right) + \beta c^n \left( 1-\frac{c^{n+1}}{N_v}\right) \right) . \end{aligned}$$

Note that this IMEX approach avoids the need of inner iterations to solve a nonlinear problem at each time step. We have used the finite difference scheme on several mesh sizes and time steps and we find that enlarging the resolution dose not yield any significant changes in the approximation for the current numerical setting (see Table 2).

### Monte Carlo Simulation

Monte Carlo simulations are widely used in many quantitative probabilistic and statistical investigations that permeates much of finance, engineering and contemporary sciences (Kroese et al. 2014). To obtain quantities of interest, such as (cumulative) probability distributions of output variables and correlations, Monte Carlo simulations enable random sampling of input parameters from predefined probability distributions and extensive repetitive experiments.

Due to the variety of viruses and variations from patient to patient, many variables can hardly be determined or measured. For instance, the dose of a virus $\gamma$ may depend on its effectiveness and toxicity, which varies among viruses. In addition, some variables, such as the concentration threshold at the time of viral infection $\hat{c}$, the reproductive rate of the virus in cancer cells $\alpha$ and human immune strength $\beta$, may all depend on patient lifestyle, gender and genetic pattern, and hence vary from patient to patient. However, the above-mentioned variables may be quantitatively correlated to viral treatment outcomes, and thereby Monte Carlo simulations are performed on $X\in \{\gamma ,\hat{c},\alpha , \beta \}$. We assume that *X* follows a normal distribution $X \sim \mathrm {N}(\mu , \sigma ^2)$ with the mean value $\mu$ and the standard deviation $\sigma$. Therefore, the stochastic variable *X* with a number of samples $N_s$ follows

20 $$\begin{aligned} X\sim \mu + \sigma \mathrm {N}(0,1). \end{aligned}$$

Taking the computational time into account, Monte Carlo simulations are performed in two-dimensional simulations. Furthermore, Monte Carlo algorithms tend to be scalable and rely less on computational dimensionality. Referring to our previous work in (Chen et al. 2018a), the accuracy of Monte Carlo simulation is proportional to the reciprocal of the square of the number of samples $N_s$, therefore 5000 samples are chosen to guarantee a small error.

## Numerical Results

To perform the simulations, the most numerical parameters have been listed in Table 2.

**Table 2:** Numerical parameters

Parameters	Notation	Value and Units
Computational domain	${\Omega }$	$15 \times 15 \times 15\,\text {mm}^3$
Lattice points	$N_l$	$30 \times 30 \times 30$
3D domain volume	Vol($\Omega$)	3375 $\text {mm}^3$
2D domain volume	Vol($\Omega$)	225 $\text {mm}^2$
Time step	${\Delta } t$	0.1 h
Mesh resolution	*h*	0.5 mm
Proliferation rate of viruses	$\beta$	1 h$^{-1}$

**Fig. 4:**
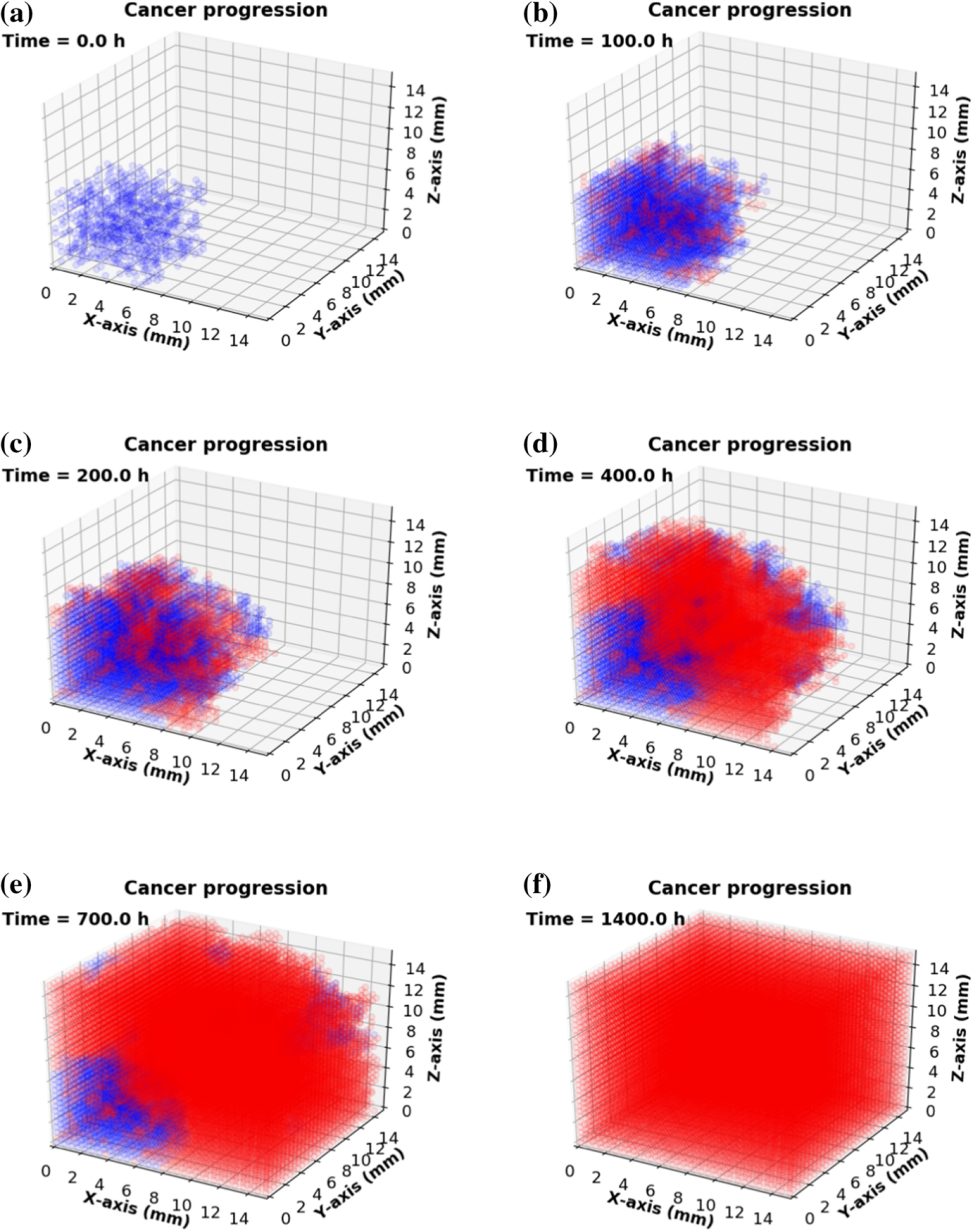
Consecutive snapshots of cancer progression, where blue color and red color are visualized as epithelial and cancer cells, respectively. The 3D domain $15 \times 15 \times 15 \text {mm}^3$ meshes into $30 \times 30 \times 30$ lattices. As a result, cancer cells occupy the entire computational domain when $t = 1400$ h.

### Cancer Progression

We consider a cubic domain to represent the tissue in the pancreas. The 3D domain ${\Omega }$ has been divided into $N_l$ lattice points. Each lattice point is occupied by multiple cells and the volume of the solid tumor *V*(*t*) at time *t* can be easily calculated by

21 $$\begin{aligned} V(t) = \frac{N_c(t)\times \mathrm {Vol}({\Omega })}{N_l^3} \ (\mathrm {mm}^3), \end{aligned}$$

where $\mathrm {Vol}({\Omega })$ is the domain volume and $N_c(t)$ denotes the number of lattice points in cancer state $\{S_i = 2\}$ at time *t*. To model cancer mutation occurring at the edge of a tissue or organ and its competitive growth with epithelial cells, a small number of lattice points are generated randomly in one octant of the domain only (see Fig. 4a). Those lattice points are initiated with epithelial cell state indicated by blue color. Due to mutation, several lattice points change their states from $\{S_i = 1\}$ to $\{S_i = 2\}$ that are visualized by the red dots (see Fig. 4a). Typically, normal cells stop dividing once they contact with each other during division as a result of contact inhibition, which can prevent excessive proliferation. Contrarily, mutated cancer cells often show uninterrupted growth that is called ‘autonomous growth’. Moreover, cancer cells disperse more easily and invade the neighborhood tissue. Therefore, they have a larger growth and division rate despite limited space and nutrient supply. In the current simulation, the probability of mutation, proliferation and death is based on Eq. (3) and several consecutive snapshots are shown in Fig. 4.

**Fig. 5:**
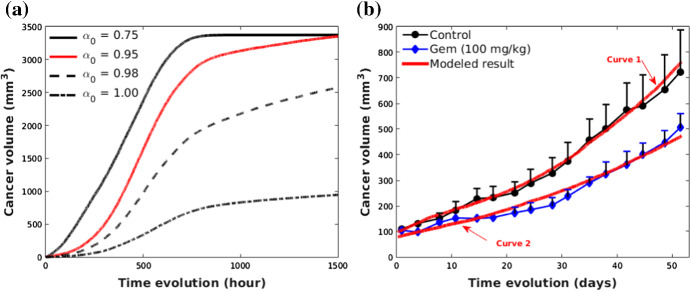
Growth curves of pancreatic tumor under different situations. **a** A comparison of growth curves of pancreatic tumors with various $\alpha _0$ value (see Eq. (6)), where $\lambda _{\mathrm {max}} = 5\times 10^{-3}$; **b** A comparison of numerical results with experimental results referring to (Durrant et al. 2015), where control and gem in the legend denote tumor growth without drug and with gemcitabine drug, respectively. In the simulation with curve 1, $\lambda _{\mathrm {max}}$ and $\alpha _0$ are equal $1\times 10^{-3}$ and 0.94, respectively. However, to calibrate the model to curve 2, $\lambda _{\mathrm {max}}$ decreases to $5.5\times 10^{-4}$ and $\alpha$ falls to 0.85

After 1400 hours (approximate 58 days), cancer cells occupy the entire computational domain and its corresponding growth curve with the respect of time indicated in red color is shown in Fig. 5a. According to Eq. (8), the growth of cancer cells is influenced by $\alpha$, which can be decided by $\alpha = \alpha _0\cdot \frac{n_1}{n_{12}}$. To investigate the impact of $\alpha _0$ on the tumor growth curve, multiple values (i.e., 0.75, 0.95, 0.98, 1) are used and the results show that growth of tumor volume slows down with the increase of $\alpha _0$ value. Therefore, small variations of $\alpha _0$ value facilitate our numerical model fitting experimental results.

As an example, Fig. 5b shows experimental results of tumor growth curve from the work by Durrant et al. (2015), where pancreatic cancer cells are inoculated into immunodeficient mice, where the inoculation site is subcutaneous. Implanted cancer cells are allowed to grow during two weeks before the initiation of gemcitabine drug treatment (${100}\,{\upmu \mathrm{g}/\mathrm{kg}}$) and its growth curve is indicated by the blue curve in Fig. 5b. As a control experiment, the black curve in Fig. 5b exhibits the growth of inoculated tumor without treatment. To mimic the tumor progression in this situation, we set up a model with a number of cancer cells initially in the domain. With minor variations of $\lambda _i$ in Eq. (4), our model is able to simulate various growth modes of pancreatic tumor (indicated by the red lines in Fig. 5b), which fits the experimental results well. Over 50 days, our numerical results regarding the increase in tumor volume show a consistency with the experimental work (Durrant et al. 2015).

### Oncolytic Virotherapy

**Fig. 6:**
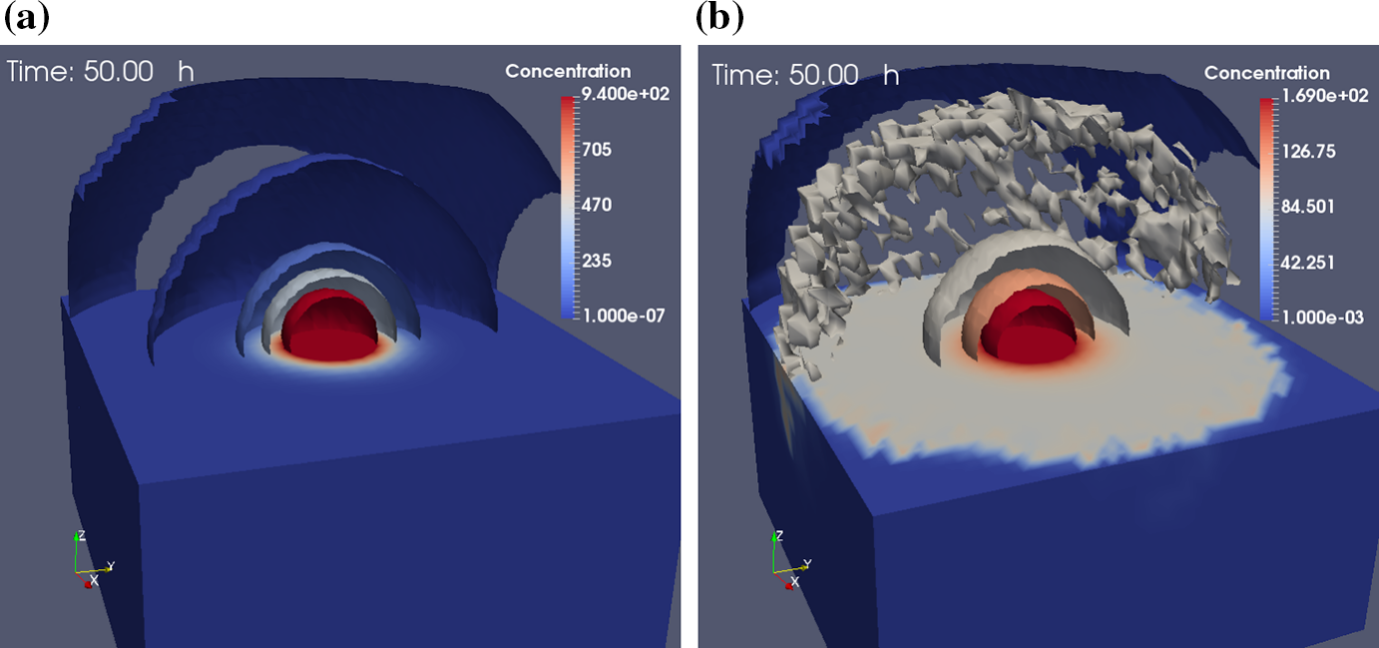
A comparison of viruses diffusion by using the FDM method with a color bar indicating the concentration of viruses, where red color represents a high concentration of virus, dark blue hints a neglectable viral concentration and other colors denote values in between. **a** No cancer cells are present and viral infection is not simulated, which means no new proliferating viruses. Therefore, most viruses are mainly concentrated in the center; **b** In the presence of cancer cells, viral infection ensues, viruses replicate leading to rupture of cancer cells, which then releases the viruses. The viruses are thus found also at distant locations. The isosurface in grey color has a concentration value of slightly less than 100 pfu $\hbox {mm}^3$

Oncolytic virotherapy has been recognized as a promising cancer treatment approach. We first develop a phenomenological model of oncolytic virotherapy in 3D, where the intratumoral injection of the virus is taken into consideration. The spread of viruses is simulated by the reaction-diffusion equation that is solved by using the FDM method. As a result, the diffusion of viruses in two different situations at time $t = 50 \ \mathrm {h}$ are compared in Fig. 6. Due to the very different doses of virus administration (Aghi and Martuza 2005; Wollmann et al. 2012), we assume that the injection is carried out during a time span of 0.5 h with a total dose of approximate $3.6\times 10^{5}$ pfu viral particles. Figure 6a shows viruses spread with the absence of cancer cells and new breeding viruses, whereas Fig. 6b gives the distribution of viruses at time $t = 50 \ \mathrm {h}$ with the viral infection and newly generated viruses. A few isosurfaces are plotted with a color bar indicating the concentration of viruses. In contrast, viruses remain in the core of the computational domain and the highest concentration of viruses is up to $9.4\times 10^{2}$ pfu $\hbox {mm}^3$ in Fig. 6a. This is mainly due to a slow viral diffusivity (Bajaj et al. 2001) and insufficient viruses supply. The isosurface in Fig. 6b indicates that a small amount of viruses has spread near the boundary. Note that irregular isosurface in grey color has a concentration value of slightly less than 100 pfu $\hbox {mm}^3$, since the new burst size of viruses from a lysed cancer cell is 100 pfu $\hbox {mm}^3$ in the current model. Due to viral infection, the highest concentration of viruses in the core is $1.69\times 10^{2}$ pfu $\hbox {mm}^3$.

**Fig. 7:**
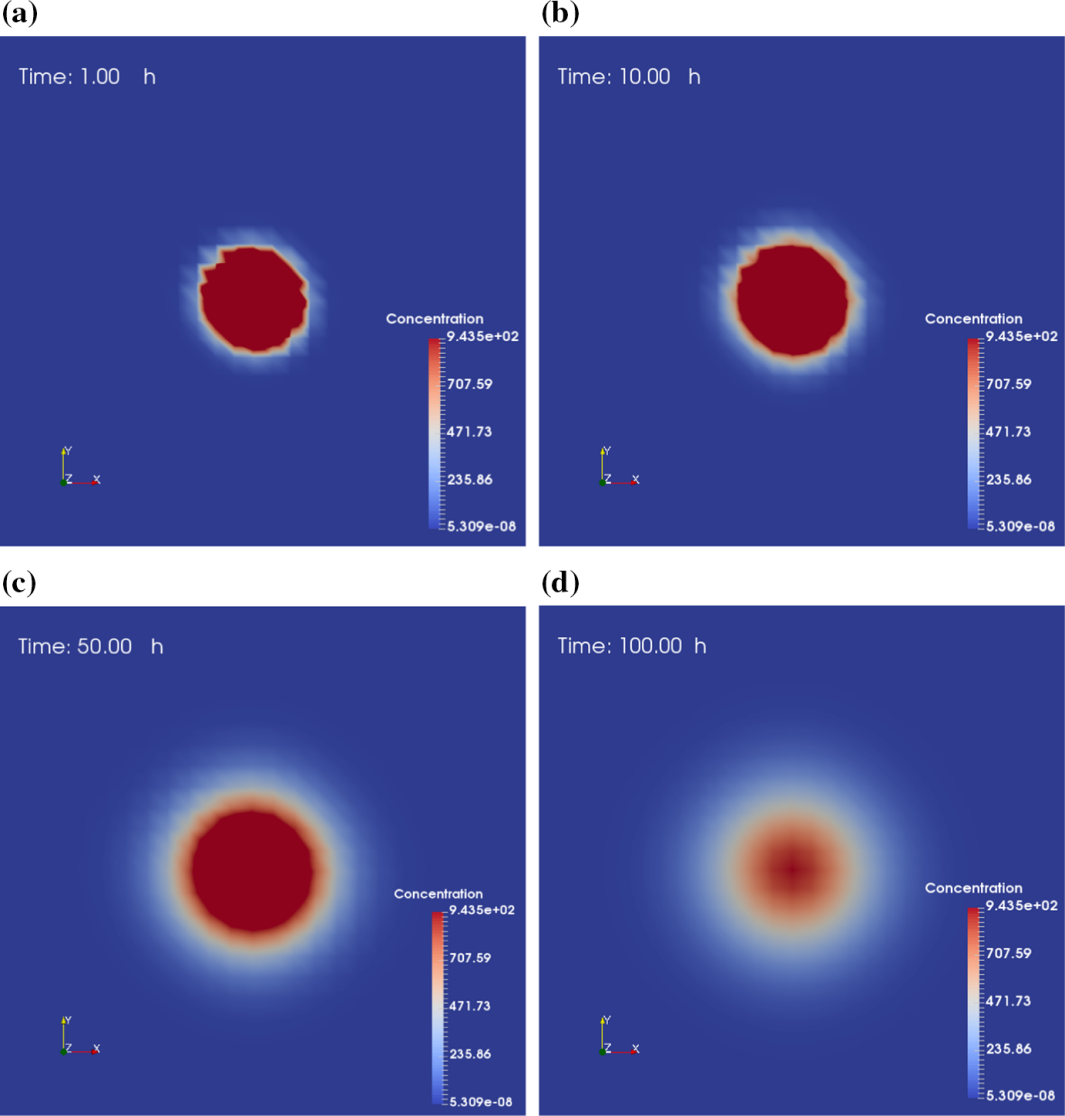
Consecutive slice plots of viral spread. No cancer cells are present and viral infection is not simulated, which means no new proliferating viruses. The slices are taken from the angle of a z-axis top view, which is located in the middle of the computational domain. A color bar indicates the concentration of viruses

**Fig. 8:**
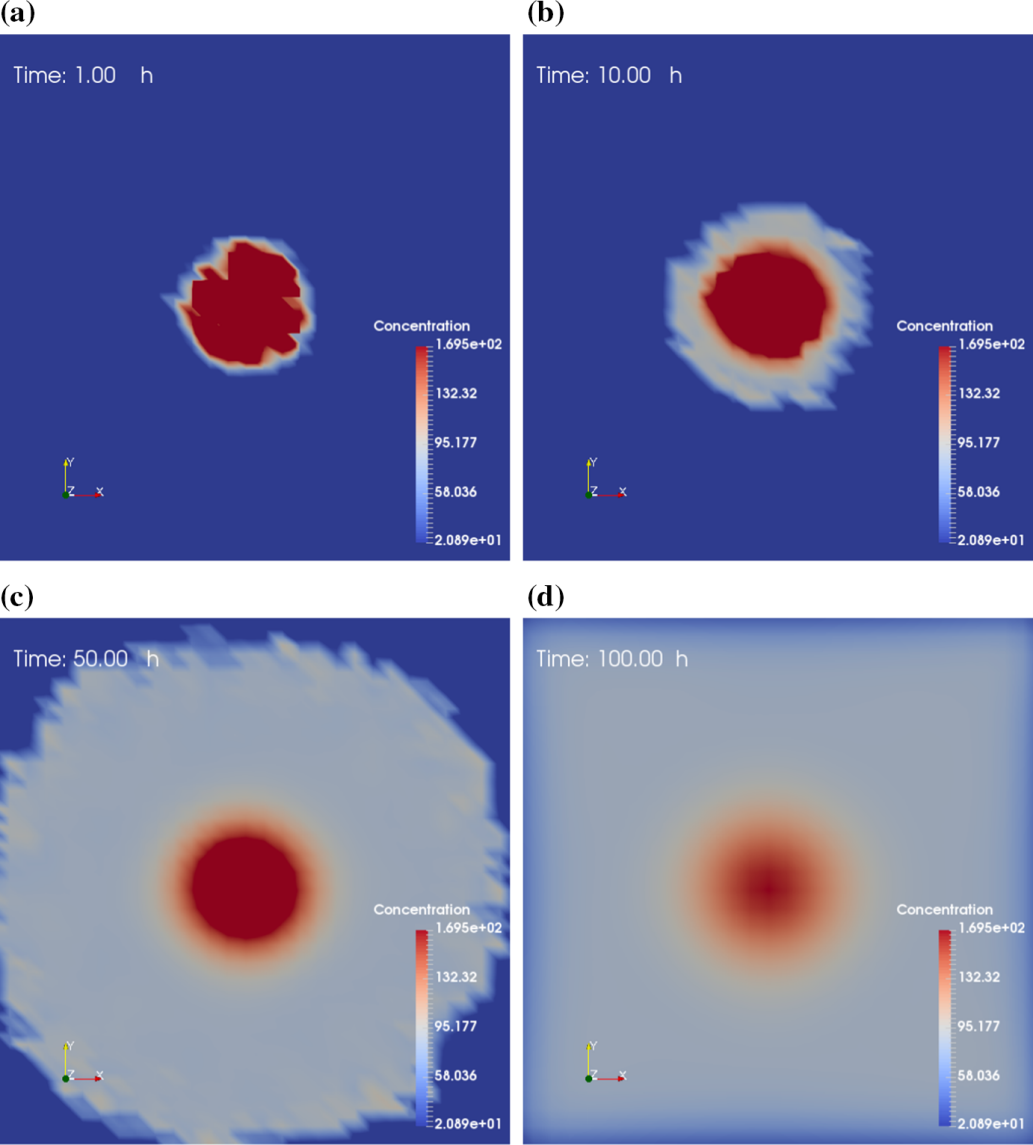
Consecutive slice plots of viral spread. In the presence of cancer cells (cancer cells are not shown for clarity), viral infection ensues, viruses replicate leading to rupture of cancer cells, which then releases the viruses. The slices are taken from the angle of a z-axis top view, which is located in the middle of the computational domain. A color bar indicates the concentration of viruses

In addition, viral diffusion with the time evolution (when $t = 1, 10, 50, 100 \ \mathrm {h}$) of each situation is shown by slices in Figs. 7 and 8, respectively. The slices are taken from the angle of a z-axis top view, which is located in the middle of the computational domain. Figure 7 presents a slow and relatively smooth diffusion phenomenon, with no viruses on the computational boundary at $t = 100 \ \mathrm {h}$. However, viral spread in Fig. 8 is faster as a result of the supply new breeding viruses from necrotic cancer cells and seems to be more random, which we think to be more in line with the infection and spread of viruses in reality. Eventually, some viruses spread to the edge and are dissipated from the border of the domain. Dissipated or remaining viruses after treatment might be removed by immune cells or, in worse cases, be virulent to healthy tissue. Therefore, it is vital to assess the toxicity of the remaining viruses after treatment.

**Fig. 9:**
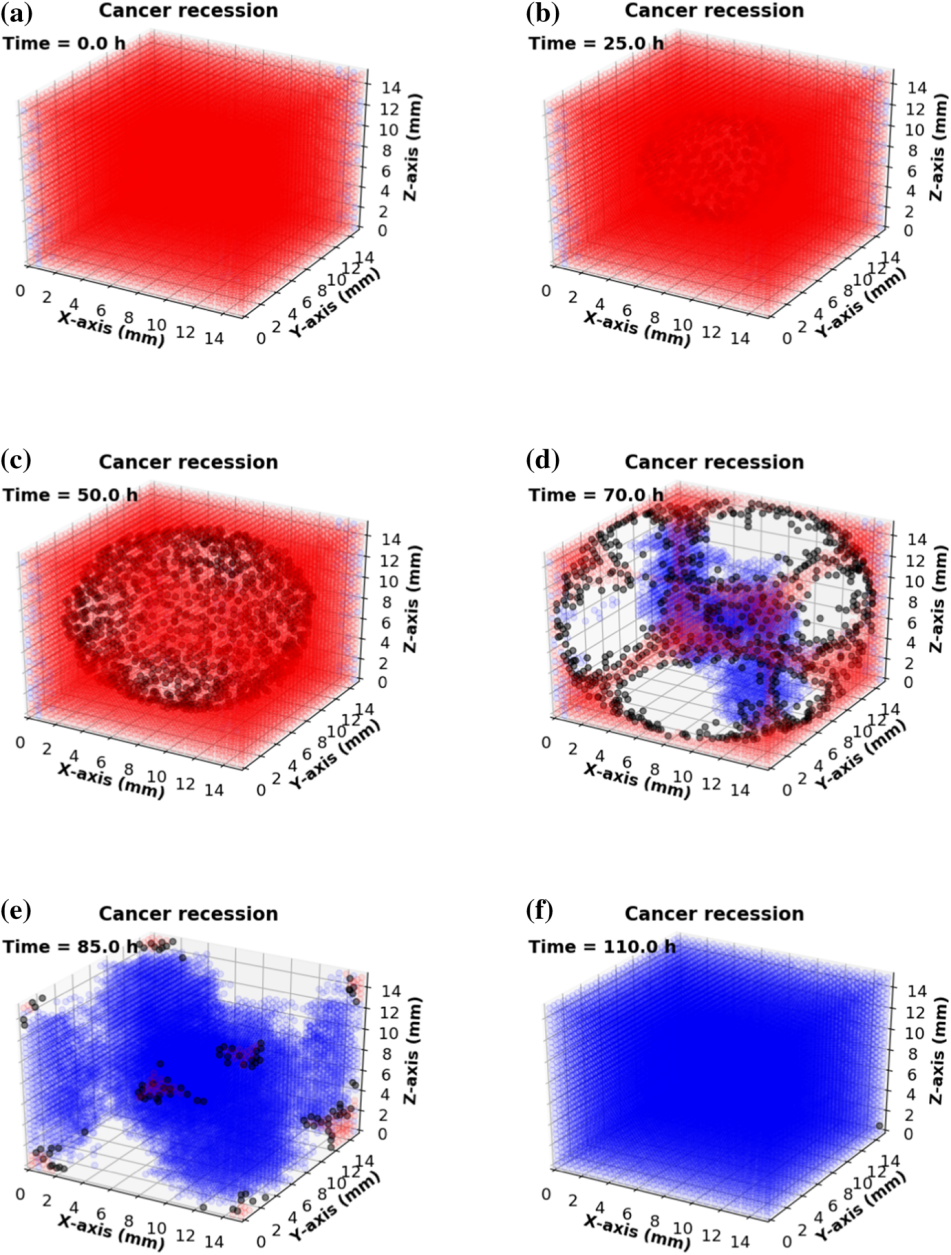
Consecutive snapshots of oncolytic virotherapy. The blue, red and black colors are visualized as epithelial, cancer and infected cancer cells, respectively. In addition, white color means the dead cells or unoccupied lattice points. A small scale of cancerous tissue that returns to normal tissue by cell reproduction or migration under the oncolytic virotherapy after $t = 110$ h

To visualize the modeling progression of oncolytic virotherapy, some consecutive snapshots are shown in Fig. 9. An extension of the model in Fig. 4, where the computational domain ${\Omega } = 15 \times 15 \times 15\,\mathrm{mm}^3$ has been divided into $N_l = 30 \times 30 \times 30$ lattices. The domain is filled with cancer cells at time $t = 0 \ \mathrm {h}$, see Fig. 9a. Typically, viruses are injected intratumorally if cancer occurs under the epidermis (Rehman et al. 2016), otherwise, intravenous injection is the main approach for virotherapy (Aghi and Martuza 2005). However, the intravenous injections could cause many viruses to infect other tissue outside of the tumor, or be removed by the immune system or be dissipated before reaching the cancer area. To make the problem tractable, we consider one dose of intratumoral injection, which is given at the center of the domain. Subsequently, internal cancer cells will start to get infected, indicated in black color, and subsequently die, which is indicated by the white color, see Figs. 9b, c. Local cells at lattice point *i* may get infected once the local concentration of viruses exceeds the threshold, which is $\hat{c} = 10\,\hbox {pfu} \,\hbox {mm}^3$. Afterward, infected cancer cells (black color in Fig. 9) are able to die (unoccupied grid nodes in white color) and release new breeding viruses with a burst size $N_s = 100\,\,\hbox {pfu} \,\hbox {mm}^3$. The dead lattice sites at state $S = 0$ are reminiscent to a wound. Then a chain reaction is triggered such that the virotherapy speeds up. Since internal lattice points are released after the death of infected cancer cells, we suppose that the normal constitutive cells around the cancer region will migrate to this area and fill the wound by proliferation, see Fig. 9d–f.

**Fig. 10:**
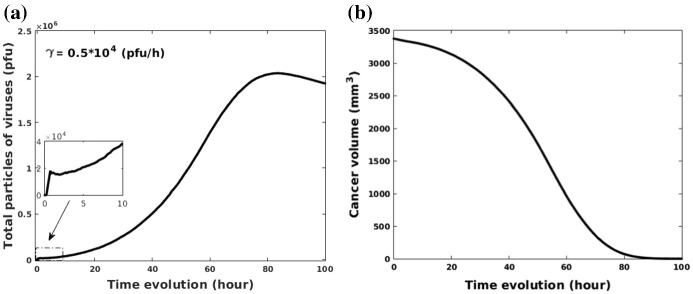
**a** Changes in viral quantity in the computational domain; **b** Changes in cancer volume with time

**Fig. 11:**
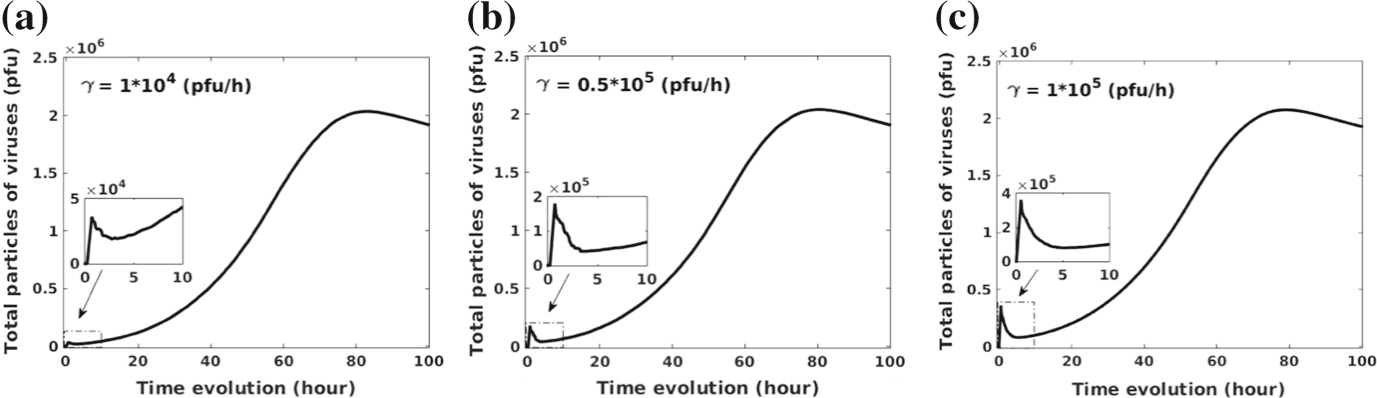
**a** Changes in viral quantity as the evolution of time with an injection rate $\gamma$ = $1\times 10^{4}$ pfu/h; **b** Changes in viral quantity as the evolution of time with an injection rate $\gamma$ = $0.5\times 10^{5}$ pfu/h; **c** Changes in viral quantity as the evolution of time with an injection rate $\gamma$ = $1\times 10^{5}$ pfu/h

The model describes an ideal virus type with a small dose to kill cancer cells, however, the role of the viral dose remains unclear. Since some viruses, like NDV, lead to a significant therapeutic benefit at high doses, whereas other viruses do not (Aghi and Martuza 2005). However, different types of viruses have different side effects (Yu and Fang 2007). The risk could be tiny symptoms, such as flu or fever (Cripe et al. 2015), and also could be severe like fatal muscle toxicity or neurotoxicity (Russell et al. 2012). Therefore, the evaluation of residual viruses after treatment is crucially important. In our model, according to the boundary condition in Eq. (9), viruses will dissipate from the boundary to other tissues or organs. Thereby we estimate the remaining viruses in the modeled area and ignore the dissipated viruses when $t = 100$ h. Figure 10a, b show changes in total viruses and cancer volume over time in the domain, respectively. At the beginning, a total dose $0.18\times 10^{5}\,{\mathrm{pfu}}$ (injection rate $\gamma$ = $0.5\times 10^{4}$ pfu/h) is given (see the enlarged view in Fig. 10a), where the domain is fully occupied by uninfected cancer cells with a volume as large as 3375 ${\mathrm{mm}^3}$ (see Fig. 10b). Once cancer cells get infected by viruses, successful viruses begin to replicate themselves until the host cancer cells rupture, which results in a significant increase in viral quantity and decrease in cancer volume. When time approaches 80 h, the number of viruses in the domain have accumulated to a peak (see Fig. 10a), whereas most cancer cells are damaged (see Fig. 10b). Furthermore, Fig. 10a shows that the number of viruses gradually decreases after 80 h and this is mainly because a fraction of viruses escapes from the domain boundary. Note that there is a minor decline in the number of viruses (see the enlarged view in Fig. 10a), which may be due to the fact that the actual number of viruses present exceeds the carrying capacity of viruses. In order to investigate whether there is a maximum capacity of viruses in a limited domain, various injection rates (i.e., $1\times 10^{4}$, $0.5\times 10^{5}$, $1\times 10^{5}$ pfu/h) are compared. The results given in Fig. 11 show that a larger viral dose leads to a greater decline in total particles after injection and do not affect the eventual result because of the maximum capacity of viruses in the computational domain. The results suggest that if a certain threshold is exceeded for the amount of injected viruses, then its temporal evolution is more or less the same.

### Monte Carlo Simulations

Kelly and Russell (2007) showed that immunosuppressed patients normally have a better therapeutic benefit than those who have an intact immune system in oncolytic virotherapy. However, a defective immune system would lead to a large number of viruses, which is associated with unacceptable toxicity in most cases (Russell et al. 2012). To make our model applicable to a wide range of virus species, the antiviral immune response is incorporated in the Monte Carlo simulations as one of the input variables, which we sample from a probability distribution. Therefore, Eq. (9) is revised slightly to

22 $$\begin{aligned} {\left\{ \begin{array}{ll} \frac{\partial c}{\partial t}- D{\Delta } c =\displaystyle \sum _{p \in \mathbb {V}(t)}\gamma \delta (\mathbf{x}-\mathbf{x}_p(t)) + \beta c (1-\frac{c}{N_v}) - \eta c, &{}\quad { \ \text {for} \ \mathbf{x} \in \ {\Omega }}\\ D\frac{\partial c}{\partial n} + T c = 0, &{} \quad \mathrm {on} \ \partial {\Gamma } \end{array}\right. }, \end{aligned}$$

The injection is modeled by Dirac delta function $\delta$(**x**) with source points at position $\mathbf{x}_p$. Moreover, $\beta$ and $N_v$ define the proliferation rate and a burst size of viruses from a necrotic cancer cell. On the domain boundary $\partial {\Gamma }$, T again represents the mass transfer rate. Note that $\eta c$ represents the neutralization process by immune cells where $\eta$ denotes the neutralization rate. Therefore, the antiviral immune strength is investigated by variation of the $\eta$ parameter. Since the appropriate dose of a specific virus is still unclear, the total dose of viral injection is considered by varying the injection rate $\gamma$.

**Table 3:** Mean and standard deviation in the Monte Carlo simulation sampling

Parameters	$\gamma$	$\hat{c}$	$\eta$
Value	($1\times 10^{4}$, ($0.4\times 10^{4}$)$^2$)	(15 5$^2$)	($1\times 10^{-2}$, ($1\times 10^{-2}$)$^2$)

**Fig. 12:**
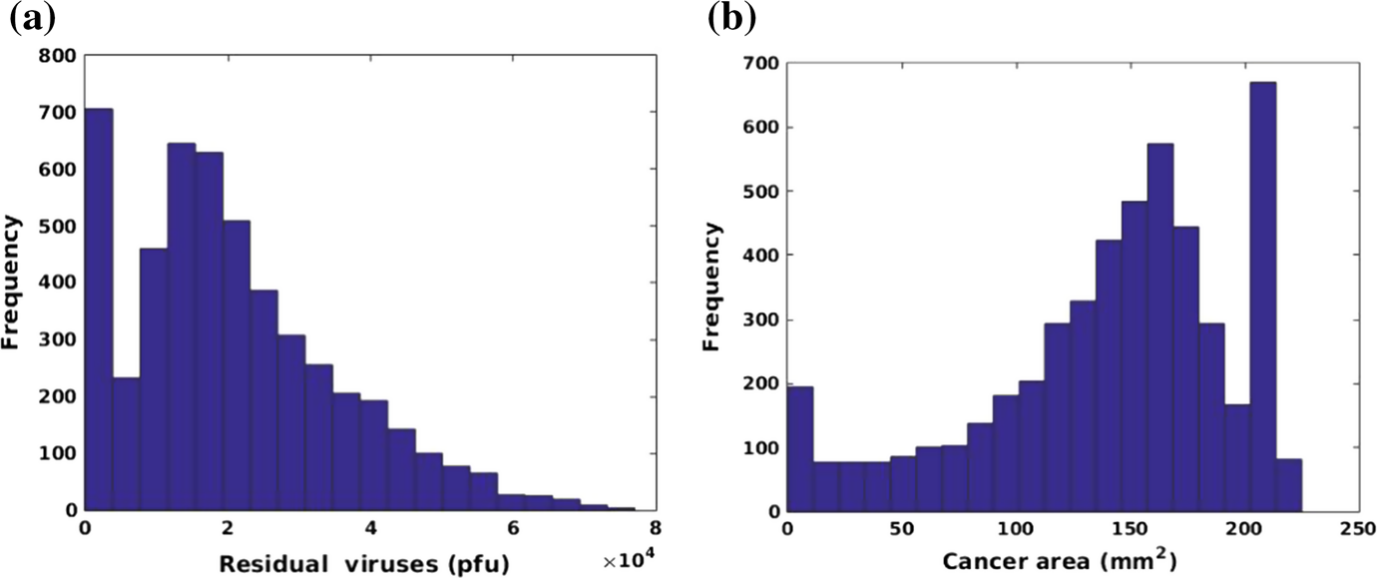
**a** Histogram of the total number of remaining particles of the viruses in Monte Carlo simulations on parameters $\gamma$, $\hat{c}$ and $\eta$; **b** Histogram of cancer area in Monte Carlo simulations on parameters $\gamma$, $\hat{c}$ and $\eta$

Moreover, the infection threshold $\hat{c}$ is used to evaluate the ability of viral infectivity regarding its impact on the final total particles of the remaining viruses and cancer area. To perform the Monte Carlo simulations, 5000 samples are chosen for the injection rate $\gamma$, infection threshold $\hat{c}$ and immune strength $\eta$, where sampling parameters follow the normal distribution. The mean and variance of the sampling parameters have been listed in Table 3. Taking CPU time into consideration, we limit each simulation up to 50 h and then compare total particles of the remaining viruses and cancer area in the computational domain. Based on 5000 samples, Fig. 12a, b show the histograms of the total particles of the remaining viruses and cancer area, respectively, in 2D simulations with a total area of the domain of 225 $\mathrm{mm}^{2}$ (15 mm $\times$ 15 mm). Of 5000 samples, 700 simulations end with few residual viruses, see Fig. 12a and thereby there are around 700 cases with a cancer area above 200 $\mathrm{mm}^2$ in Fig. 12b. This may be caused by a combination of low injection rate, high infection rate and a strong antiviral immune response.

**Fig. 13:**
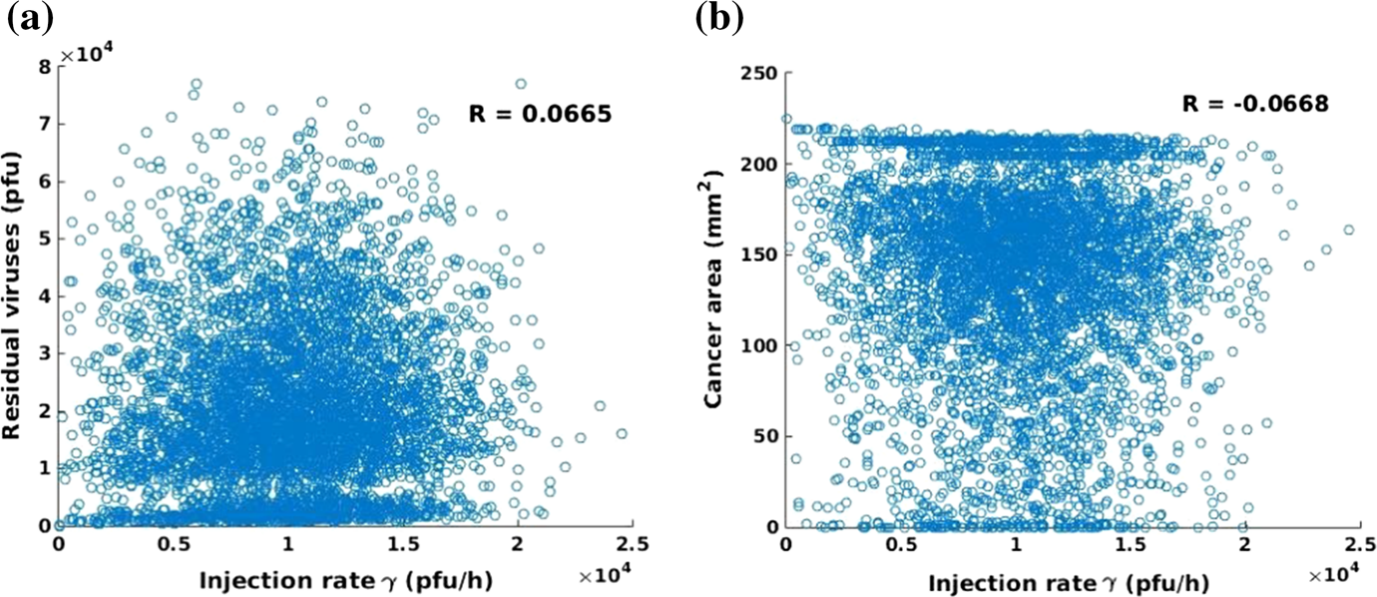
**a** Scatter plot of injection rate $\gamma$ and total particles of the remaining viruses. The corresponding correlation coefficient is $R = 0.0665$; **b** Scatter plot of injection rate of $\gamma$ and the final cancer area with a correlation coefficient $R = -0.0668$

**Fig. 14:**
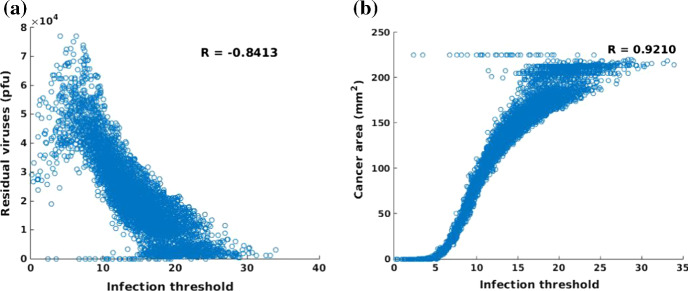
**a** Scatter plot of infection threshold $\hat{c}$ and the total particles of the remaining viruses. The corresponding correlation coefficient is $R = -0.8413$; **b** Scatter plot of infection threshold $\hat{c}$ and the final cancer area. The correlation coefficient of infection threshold $\hat{c}$ and the final cancer area is $R = 0.9210$

**Fig. 15:**
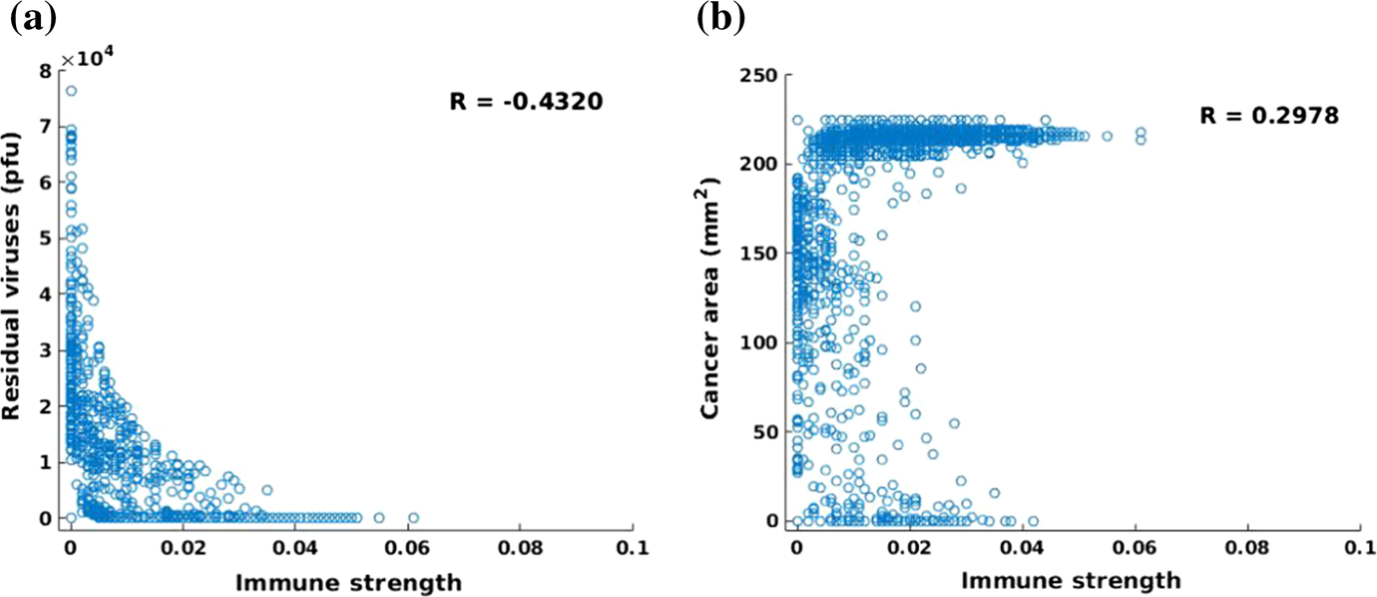
**a** Scatter plot of immune strength and the remaining viral quantity. The correlation coefficient of immune strength and the remaining viral quantity is $R = -0.4320$; **b** Scatter plot of immune strength and the final cancer area. The correlation coefficient of immune strength and the cancer area is $R = 0.2978$

Since the simulation is limited to 50 h, most cases end with a large cancer area compared with the original area, which is from 100 to 200 $$\mathrm{mm}^{2}$$. Correspondingly, there is a large portion of simulations that have the remaining viral quantity ranging from 1E$$^4$$ to 3E$$^4$$$$\mathrm {pfu}$$ at $$t = 50$$ h. To see the correlations between variables and the numerical results, several scatter plots are shown in Figs. 13, 14 and 15. The role of viral dose is tested by using the injection rate $$\gamma $$ in Fig.13, which shows that there is no obvious correlation between the injected virus dose and the remaining viral quantity and cancer area. This is probably because of an insufficient simulation time period or the maximum capacity of viruses in a limited domain (see Fig. 11 as an illustration for this claim). Since the viral particles are not necessarily contained in the domain of computation, but are leaving the considered region as a result of diffusion (possibly also as a result of being transported by the blood vessel network). The ‘loss’ of particles is modeled by the Robin boundary condition. This is the reason why that once all cancer cells have been neutralized, like happens in some of the Monte Carlo simulations, the viral particles concentration will tend to zero in the computational domain, and hence a flattening behavior takes place along the ‘no residual viruses axis’, see Fig. 13a. The flattening behavior that is shown in Fig. 13b reflects the Monte Carlo runs for cases that the viral particles are already transported away from the domain of computation and hence the cancer cells are not reached by which the viral doses are not able to reproduce sufficiently and hence the entire region ($$15 \times 15 \times 15 \mathrm {mm}^2 = 225 \mathrm {mm}^2$$) remains full with cancer cells.

In contrast, the infection threshold, which is used to represent the ability of viral infectivity, shows a significant correlation with the remaining viral quantity and cancer area in Fig. 14. The higher the threshold value, the higher the concentration of the virus is needed to infect the cancer cells, which hints at a lower ability of the viral infectivity. From Fig. 14a, the number of residual viruses decreases with increasing infection threshold since viruses with low infection ability are not able to damage cancer cells, but they can be eliminated by the antiviral immune response. Therefore, antiviral immune and insufficient newborn viruses facilitate cases with few residual viruses after 50 h. Certainly, the cancer area will not reduce significantly if the viral infection ability is weak. Viruses with a good infectivity ($\hat{c} \le 5$) are able to neutralize cancer cells within a period of $t = 50$ h. Based on Eq. (22), the term $\eta c(\mathbf{r})$ reflects the immune strength, therefore, the immune strength is investigated through variation of immune reduction rate $\eta$. Note that the value of $c(\mathbf{r})$ is quite large and thereby the $\eta$ is chosen very small (from 0 to 0.06) to guarantee a simulation with a likelihood of success. A large $\eta$ denotes a strong antiviral immune response that would result in the death of most viruses. According to Fig. 15a, in the case of immunodeficiency, the residual viruses could accumulate to a large amount, while the amount falls as the immunity increases. When the antiviral response is strong like $\eta > 0.04$, viruses will be eliminated by immune cells completely in the domain. On the contrary, the cancer area has declined with the intervention of residual viruses if the antiviral immune is defective (see when $\eta$ approaches to 0). However, the cancer area is more likely to be large in size when the immune response is strong, like $\eta > 0.04$. This indicates that patients with a weaker immune response may benefit from a larger reduction of the tumor size. However, at the same time, patients with a weak immune system are sensitive to have large amounts of residual viruses in their bodies.

## Conclusions

Many animal-based experiments and clinical trials yielded a noticeable tumor attenuation by using oncolytic viruses (De Pace 1912; Kasuya et al. 2005). However, currently, viruses are not deemed as a useful means to stop or inhibit cancer since there is no effective way to control the virulence and retaining their replication capability in cancer cells (Kelly and Russell 2007). We have developed a cell-based model in pancreatic cancer at early stages (Chen et al. 2018b), which is subsequently extended to therapy model in (Chen et al. 2019). However, compared to classical treatments for pancreatic cancer like surgery, radiotherapy, chemotherapy, virotherapy has its own limitations, which needs further scientific assessment. In particular, the limitations include antiviral immune responses, inefficient delivery of virus as well as the poor virus spread in tumor area (Wennier et al. 2011). Therefore, more research in terms of oncolytic virotherapy is needed.

In the present study, we develop a 3D cellular automata model for oncolytic virotherapy. As we expected, the model is able to simulate cancer progression at early stages, which include the biological processes such as mutation, proliferation and death. Within 1400 h (appropriate 58 days), cancerous cells mutate from healthy somatic cells and then colonize the computational domain as big as $15 \times 15 \times 15 \ \mathrm {mm}^3$. Certainly, the model is scalable and the speed of cancer progression can be adjusted by variation of input parameters. Therefore, different growth trends have been compared and one numerical result of our model could fit experimental results very well. Subsequently, oncolytic virotherapy is phenomenologically simulated in the same domain that is completely occupied by cancer cells. The migration and proliferation of the virus is modeled by using a reaction-diffusion equation, which is solved by a FDM method. Since viruses specifically infect and damage cancer cells, the model predicts cancer attenuation as time evolves. Eventually, normal somatic cells fill in the gap through migration and proliferation.

In addition, Monte Carlo simulations are performed in a 2D model to quantitatively investigate the correlations between several input variables and numerical results. Among 5000 samples, there are 700 simulations ending with few residual viruses and large cancer area, which dues to failed virotherapy probably as results of the extreme parameter values. The results indicate an insignificant correlation between the injection dose of viruses and simulated results (total residual viruses and cancer area), and that is probably because of an improper value range of injection rate, an insufficient simulation time period or a limited computational domain. However, we believe that this result is acceptable, since some virus species, such as NDV, show a high correlation between given doses and therapeutic benefits, whereas others do not (Aghi and Martuza 2005). Further, the viral infection threshold has a significant correlation with total amount of remaining viruses and with the final cancer area, which means that viruses with low viral infectivity likely allow a large cancer area with just few viruses left. Moreover, the anti-viral immune response presents an obvious correlation with the numerical results. Specifically, most simulations end up with relatively fewer viruses if the anti-viral reaction is strong and thereby the corresponding residual cancer area is also larger.

Due to gene mutation (i.e., RAS, TP53), the anti-viral infection ability of cancer cells is weakened, which gives the oncolytic viruses a chance (Chiocca 2002) to infect the cancer cells. Research in oncolytic viruses is not limited to cancer therapy research, also in studies that combine other treatments, such as immunotherapy (Bommareddy et al. 2018) and chemotherapy (Molnar-Kimber et al. 2002). In order to optimize the viral therapy in terms of fighting cancer, and leaving as few viral particles post-therapy as possible, further experimental studies are necessary. The required quantification in order to optimize viral therapy implies that mathematical modeling is a necessary and very helpful step.
